# Giant Coronary Artery Aneurysms Presenting As Posterior Myocardial Infarction

**DOI:** 10.7759/cureus.52081

**Published:** 2024-01-11

**Authors:** Syed M Ishaq, Sanchit Duhan, Bijeta Keisham, Taha Khalid, Badr Harfouch

**Affiliations:** 1 Internal Medicine, Sinai Hospital of Baltimore, Baltimore, USA; 2 Internal Medicine, Weifang Medical University, Weifang, CHN; 3 Medicine, Services Hospital, Services Institute of Medical Sciences, Lahore, PAK; 4 Cardiovascular Medicine, Sinai Hospital of Baltimore, Baltimore, USA

**Keywords:** giant coronary artery aneursym, coronary artery ectasia (cea), acute coronary syndrome, myocardial infarction, coronary aneurysm

## Abstract

A coronary artery aneurysm (CAA) is defined as the dilatation of a vessel with a diameter of ≥1.5 times that of the adjacent normal vessel. Occasionally, aneurysms can be large enough to be characterized as giant CAAs, but there is no universally accepted definition. We discuss the case of a 45-year-old male patient who presented to the hospital with substernal chest pain. His ECG revealed ST depression and T wave inversions in precordial leads. Cardiac biomarkers were within normal limits. Due to concerns about coronary artery disease, cardiac catheterization was done, which revealed CAAs in the distribution of the right coronary artery (RCA), left anterior descending (LAD) and left circumflex (LCX) artery. The patient was at high risk for surgical intervention given coexisting severe pulmonary hypertension. Therefore, medical treatment was initiated with beta-blockers, high-intensity statin, and anticoagulation with warfarin. In a two-month follow-up, the patient remained asymptomatic without any residual symptoms. A CAA can present as an acute coronary syndrome. The treatment still evolves, involving medical management and/or percutaneous or surgical interventions.

## Introduction

An abnormal widening of a specific portion of the vessel wall identifies aneurysmal coronary artery disease [1]. Coronary artery aneurysm (CAA), also known as coronary artery ectasia (CEA), is defined as a dilatation of a vessel with a diameter of ≥1.5 times that of the adjacent normal vessel [2]. On occasion, CAAs can be large enough to be characterized as giant coronary artery aneurysms. There is no universally accepted definition of giant CAAs. The literature proposes diameters greater than 20 mm, 40 mm, and 50 mm and quadruples the reference vessel diameter [3]. A CAA is most commonly present in the proximal and middle segments of the right coronary artery (RCA) (68%), followed by the proximal left anterior descending artery (LAD) (60%), and the left circumflex (LCX) arteries (50%) [1]. A CAA of the left main stem is rare and occurs only in 0.1% of the population [4]. Coronary artery aneurysms are mostly atherosclerotic in origin. However, they could also be mycotic, congenital, or present as a part of systemic inflammatory disorders such as polyarteritis nodosa or Kawasaki disease [1]. The overall incidence of CAA in patients undergoing coronary angiography is reported to be 0.3%-4.9% and 2%-10% after an intervention procedure [5]. The incidence of giant CAA is even lower at 0.02% in the patients who undergo coronary angiography, although the number depends on the size definition [6]. We present a rare case of a giant CAA (>20 mm in diameter).

## Case presentation

A 45-year-old male patient presented to the emergency department with a chief complaint of substernal chest pain. He stated that he developed a sudden onset of substernal chest pain accompanied by nausea, vomiting, and diaphoresis. The patient had a medical history of severe pulmonary hypertension, obstructive sleep apnea, schizophrenia, polycythemia, and right upper extremity deep venous thrombosis (currently taking rivaroxaban).

On a physical exam, the patient appeared to be in a moderate amount of distress, with substernal chest pain without any radiation. His baseline laboratory workup, including a complete blood count and comprehensive metabolic panel, was within normal limits (Table 1).

**Table 1:** Laboratory work-up on admission AST: aspartate aminotransferase; ALT: alanine aminotransferase; WBC: white blood cells

Laboratory panel	Results on admission	Normal range
Sodium	140 mmol/L	135-145 mmol/L
Potassium	4.1 mmol/L	3.5 - 5.1 mmol/L
Chloride	104 mmol/L	98 - 107 mmol/L
Creatinine	0.99 mg/dl	0.50 - 1.30 mg/dL
Calcium	9.4 mg/dl	8.5 - 10.1 mg/dL
Magnesium	1.9 mg/dl	1.8 - 2.6 mg/dL
AST	30 unit/L	8 - 33 unit/L
ALT	17 unit/L	4 - 36 unit/L
Alkaline phosphatase	49 unit/L	45 - 117 unit/L
Total bilirubin	0.80 mg/dl	0.1 - 1.2 mg/dl
WBC count	4.47k cells/uL	4.5 - 11.0k cells/uL
Hemoglobin	15 gm/dl	13.2 - 18.0 gm/dl
Platelet count	160,000	150,000 - 450,000 cells/uL

Also, his pro-brain natriuretic peptide (BNP) and two sets of troponin labs were normal (Table 2).

**Table 2:** Cardiac enzymes and biomarkers on admission Pro-BNP: pro-brain natriuretic peptide

Laboratory panel	10:00 (Day 1)	15:00 (Day 1)	Normal range
Troponin	12 ng/L	13 ng/L	<14 ng/L
Pro-BNP	81 pg/mL	-	<125 pg/mL

**Figure 1:**
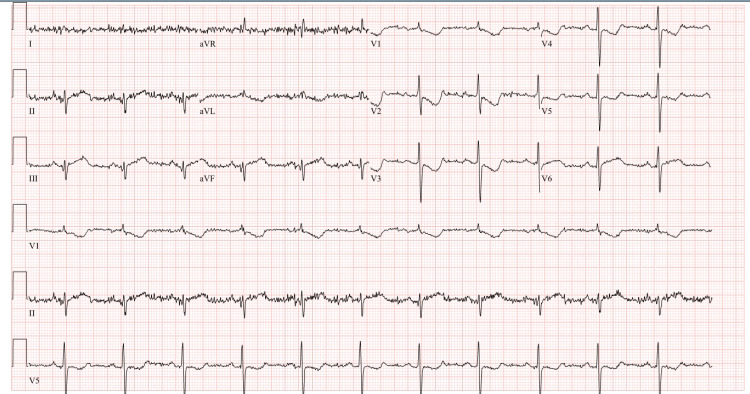
Electrocardiogram on admission Deep T wave inversions in the precordial leads V1 to V5 without reciprocal ST changes.

His initial electrocardiogram (ECG) revealed deep T wave inversions in the precordial leads V1 to V5 without any reciprocal ST wave changes (Figure 1). 

**Figure 2:**
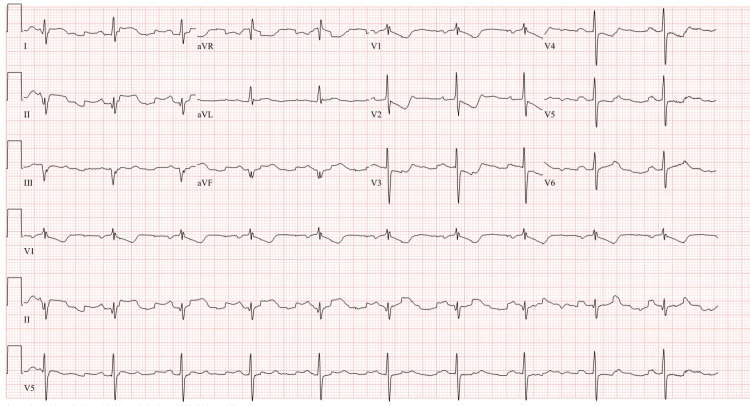
Repeat electrocardiogram performed due to persistent chest pain Deep T wave inversions in the precordial leads V1 to V5 with reciprocal ST depression in leads V1 and V2.

The patient was treated with aspirin and morphine. However, the pain remained persistent without any resolution, and another ECG was obtained, which revealed ST depressions in lead V1 and V2 and persistent T wave inversions (Figure 2).

Due to concerns for non-ST elevation acute coronary syndrome, the patient was urgently taken for a cardiac catheterization.

**Figure 3:**
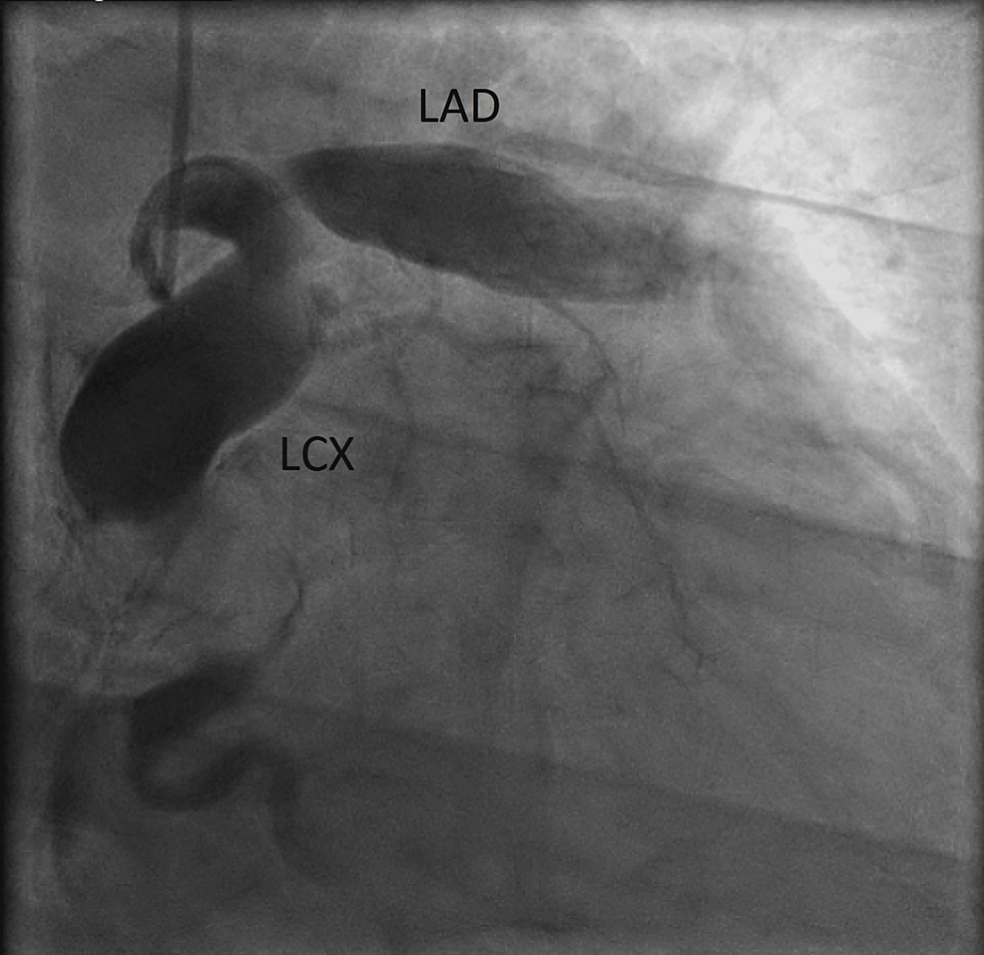
Left heart catheterization in RAO cranial view demonstrating aneurysmal dilatation in the left anterior descending artery and left circumflex. RAO: right anterior oblique; LAD: left anterior descending artery; LCX: left circumflex

A left heart catheterization revealed a giant CEA in the left anterior descending, left circumflex, and right coronary artery distribution. The aneurysm diameters were found to be 11 mm in the proximal LAD artery and 14 mm in the proximal LCX artery (Figure 3).

**Figure 4:**
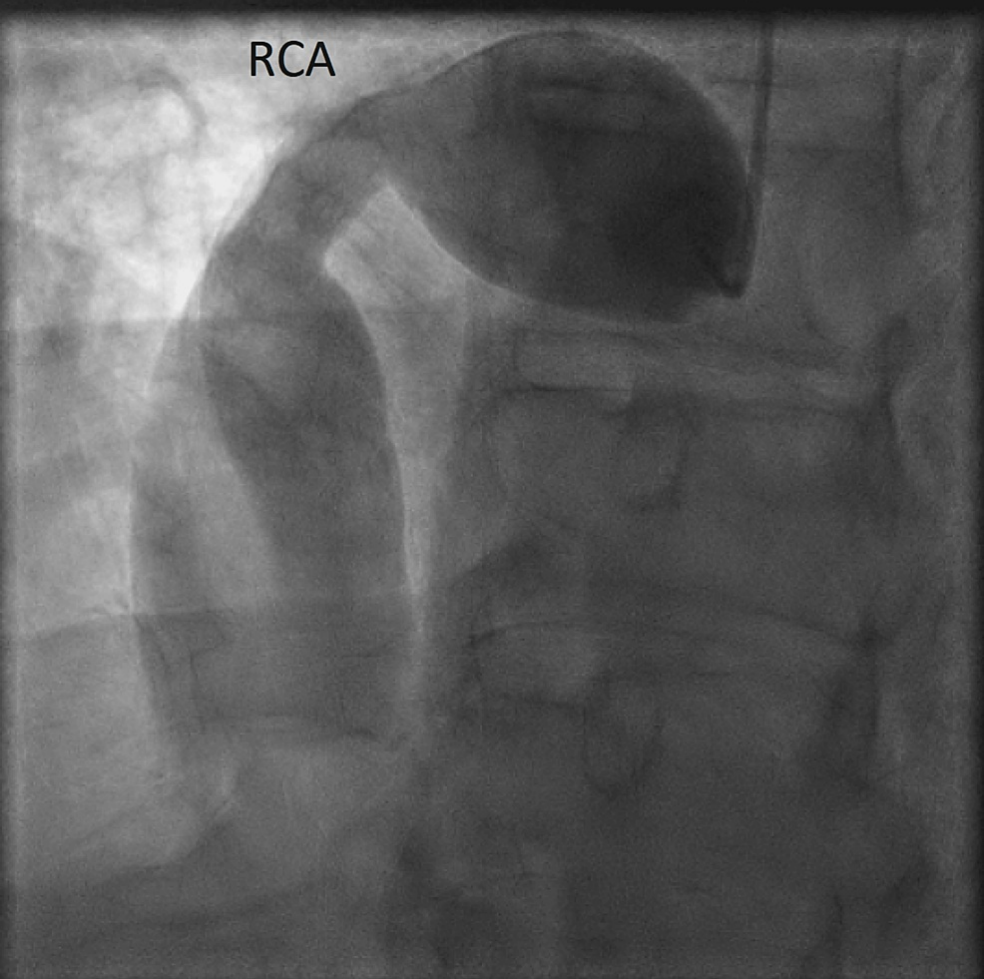
Left heart catheterization in LAO cranial view demonstrating aneurysmal dilatation in the proximal and mid portion of the RCA LAO: left anterior oblique; RCA: right coronary artery

The aneurysm diameters appeared to be 24 mm and 14 mm in the proximal and mid-distribution of the RCA (Figure 4).

The distal portion of the arteries could not be visualized. The LCX artery revealed a filling defect in the mid portion concerning stenosis. However, the thrombotic obstruction in the LCX was not amenable to intervention. The left ventricle end-diastolic pressure was 14 mmHg, and the left ventricular ejection fraction was more than 55%.

Due to the significant diameter of the aneurysms, cardiothoracic surgery was consulted, and the surgeon deemed the patient at very high risk for surgery because of coexisting severe pulmonary hypertension. The decision was thus made to pursue medical management with beta-blockers, high-intensity statins, and anticoagulation. He was started on intravenous heparin with the intention to bridge and subsequently initiate warfarin with close follow-up.

In the two-month outpatient follow-up, the patient did not demonstrate any residual chest pain. He was able to resume his usual daily activities without any limitations. He could walk on level ground, climb a few stairs, and do household chores, depicting metabolic equivalents (METS) of four. There were no reported symptoms of dizziness, lightheadedness, or shortness of breath. There was no reported personal or family history or connective tissue disorder.

## Discussion

The true incidence of giant CEAs might be underestimated, as the thrombi can conceal the artery’s actual diameter on an angiogram. Coronary artery ectasias are most common in Asian and Hispanic patients and least common in patients of African American ethnicity [7, 8]. The clinical presentation can be completely asymptomatic or present with acute coronary syndrome, angina, or dyspnea [9]. Giant CEAs are primarily symptomatic and mimic a mediastinal or cardiac tumor [10]. The underlying pathophysiologic mechanism is unclear. A genetic predisposition has been proposed. A specific allele of the matrix metalloproteinases (MMPs), MMP3-5A, is significantly more prevalent [11]. Other than atherosclerosis and coronary intervention, giant CEAs are associated with Kawasaki disease, Takayasu arteritis, connective tissue disorders (Marfan’s syndrome, Ehlers-Danlos syndrome, fibromuscular dysplasia, neurofibromatosis), vasculitis (lupus, polyarteritis nodose, Behçet's disease, rheumatoid arthritis, ankylosing spondylitis, scleroderma), infections (human immunodeficiency virus, bacterial, mycobacterial, syphilis, Lyme disease, mycotic aneurysm, septic emboli), drugs (cocaine, amphetamine, protease inhibitors), chest trauma, tumor, and cardiac lymphoma [9].

Coronary angiography is the gold standard for the diagnosis of CEAs. It can provide information regarding anatomy and associated coronary artery disease. However, it can underestimate the aneurysm size in the case of intraluminal thrombi. A multislice computed tomography coronary angiography has ~100% sensitivity and can capture complex anatomy and detect intramural thrombi. Further, a three-dimensional reconstruction helps understand the relationship between aneurysms and surrounding structures [12]. The echocardiogram has high sensitivity and specificity for evaluating the proximal left main coronary artery and right coronary artery [13]. Coronary artery ectasias can lead to complications such as fistula, cardiac compression, tamponade, congestive heart failure, or superior vena cava syndrome. There is a very high risk of thrombi in aneurysms >5mm [9]. In a large multicentric registry-based study, the mortality and major adverse cardiac events (MACE) rates associated with CEAs were 15.3% and 31%, respectively [14].

Given the lack of randomized trials, there are no established guidelines for managing CEAs, and current management is based on anecdotal reports. Medical management can be considered in small CEAs in distal locations with a low risk of complications. It consists of antiplatelet therapy and statins. Other treatments, such as anticoagulation, beta-blockers, and angiotensin-converting enzyme inhibitors, can be considered [9]. In patients with a high risk of complications, such as large CEAs in proximal locations, medical therapy alone might be insufficient. These patients are often treated with percutaneous coronary intervention (PCI) with coil embolization or covered stent exclusion or surgery (aneurysm ligation, resection, marsupialization with interposition graft, aneurysmectomy, or thrombectomy with bypass grafting) [15]. The PCI of CEAs is associated with lower procedural success, a higher risk of embolization, and higher intermediate-term mortality [16]. The success rate of surgery is unknown, given the rarity of the aneurysm.

## Conclusions

The case highlights a rare incidence of multi-vessel giant CAAs. The patient’s presentation mimicked a myocardial infarction. Even though the CAAs were giant, there were no compressive symptoms or complications. The patient was not a surgical candidate, and symptoms improved with medical management. This case adds value to the limited existing literature regarding treating giant CAAs. 
